# Effects of Sodium Bicarbonate on High-Intensity Endurance Performance in Cyclists: A Double-Blind, Randomized Cross-Over Trial

**DOI:** 10.1371/journal.pone.0114729

**Published:** 2014-12-10

**Authors:** Florian Egger, Tim Meyer, Ulf Such, Anne Hecksteden

**Affiliations:** Institute of Sports and Preventive Medicine, Saarland University, Saarbrücken, Germany; Texas A&M University, United States of America

## Abstract

**Background:**

While the ergogenic effect of sodium bicarbonate (BICA) on short-term, sprint-type performance has been repeatedly demonstrated, little is known about its effectiveness during prolonged high-intensity exercise in well-trained athletes. Therefore, this study aims to examine the influence of BICA on performance during exhaustive, high-intensity endurance cycling.

**Methods:**

This was a single-center, double-blind, randomized, placebo-controlled cross-over study. Twenty-one well-trained cyclists (mean ± SD: age 24±8 y, BMI 21.3±1.7, VO_2peak_ 67.3±9.8 ml·kg^−1^·min^−1^) were randomly allocated to sequences of following interventions: oral ingestion of 0.3 g·kg^−1^ BICA or 4 g of sodium chloride (placebo), respectively. One h after ingestion subjects exercised for 30 min at 95% of the individual anaerobic threshold (IAT) followed by 110% IAT until exhaustion. Prior to these constant load tests stepwise incremental exercise tests were conducted under both conditions to determine IAT and VO_2peak_. Analysis of blood gas parameters, blood lactate (BLa) and gas exchange measurements were conducted before, during and after the tests. The main outcome measure was the time to exhaustion in the constant load test.

**Results:**

Cycling time to exhaustion was improved (p<0.05) under BICA (49.5±11.5 min) compared with placebo (45.0±9.5 min). No differences in maximal or sub-maximal measures of performance were observed during stepwise incremental tests. BICA ingestion resulted in an increased pH, bicarbonate concentration and BLa before, throughout and after both exercise testing modes.

**Conclusion:**

The results suggest that ingestion of BICA may improve prolonged, high-intensity cycling performance.

**Trial Registration:**

German Clinical Trials Register (DRKS) DRKS00006198.

## Introduction

During prolonged cycling the ability to sustain a high power output is limited by the resources of the athlete to resist fatigue. To date, the cause of fatigue is controversial and performance may be limited by numerous factors, including physiological and psychological components [1]. However, for high-intensity exercise perturbations in both blood and muscle acid-base balance are associated with a decline in work rate and performance [2]–[4]. Thus, it has been assumed that muscle fatigue occurring in high-intensity exercise is mainly caused by the accumulation of hydrogen ions (H^+^) in the myoplasm [2], [5]. The cellular mechanisms include detrimental effects on myofilament interaction [6], glycolytic flux [4] and sarcoplasmatic reticulum function [7]. The ability of the body to prevent or delay these force limiting processes is determined by the capacity of its intrinsic buffering systems [5], which counteract the accumulation of H^+^ both inside and outside the cell [8].

Supplementation of alkalizing agents such as sodium bicarbonate (BICA) indirectly affects intramuscular pH by increasing the H^+^ concentration gradient across the sarcolemma [9]. As a result, the co-transport of H^+^ and lactate from the myoplasm is enhanced [10]. These findings suggest that increased extracellular buffering capacity may influence muscle function and potentially improve exercise performance during continuous cycling.

Consequently, BICA has been studied as a potential ergogenic substance in several trials, mainly with a focus on short-term, sprint-type exercise. In a recently published meta-analysis it was concluded that BICA can be used as an ergogenic aid in short-term, high-intensity exercise [11]. By contrast, only few studies investigated the ergogenic potential of BICA in prolonged, high-intensity exercise with controversial findings [12]–[16]. These studies are based on small sample sizes and, in part [14], [15], on subjects who are not habituated to the applied exercise mode. This is despite the relevance for typical competition modes (especially in cycling) and the lower variability of performance in trained subjects. Therefore the aim of this study was to investigate the effects of BICA supplementation on performance during prolonged, high-intensity cycling to exhaustion in well-trained athletes.

## Materials and Methods

### Subjects

Participants were recruited between May 2011 and October 2011 through email. Twenty-one healthy, well-trained cyclists (16 male, 5 female; mean ± SD: age 24±8 y, BMI 21.3±1.7, VO_2peak_ 67.3±9.8 ml·kg^−1^·min^−1^) met the following criterion for inclusion in the study: a maximal performance during a stepwise incremental cycle test of at least 4.5 W·kg^−1^ for females and 5.0 W·kg^−1^ for males, respectively. The sample consisted of nine racing bikers, nine mountain bikers and three triathletes. On their first visit to the laboratory each subject received a medical check-up consisting of 12-lead ECG, blood pressure measurement, whole-body plethysmography and blood analysis (routine blood count and clinical chemistry). During the test period subjects were instructed to continue their normal training routine but to refrain from exercise, alcohol and caffeine the day before each trial.

All subjects reached the age of majority (at least 18 years) and were fully informed of the experimental procedures and gave written informed consent prior to participation. The study was carried out in accordance with the declaration of Helsinki and approved by the local ethics committee (Aerztekammer des Saarlandes, Saarbruecken, Germany; approval number: 35/11).

Reasons for not registering this study before enrolment of participants started were low subject number and ready recruitement of subjects from a local pool of athletes. The authors confirm that all ongoing and related trials for this intervention are registered. The protocol for this trial and supporting CONSORT checklist are available as supporting information; see S1 and S2 Protocols and S1 Checklist. In addition, all relevant data are presented in tabular form; see S1 Data.

### General Design

**Figure 1:**
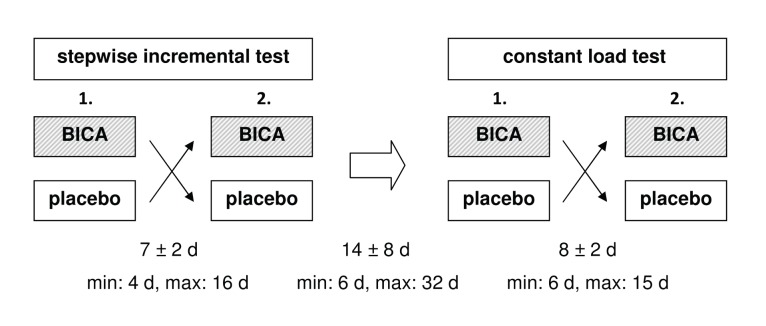
Schematic representation of the general design. Both stepwise incremental tests and constant load tests were conducted under two conditions: either after oral ingestion of 0.3 g·kg^−1^ sodium bicarbonate (BICA) or 4 g of sodium chloride (placebo). Time interval between tests is specified in days (d). Data are presented as means ± standard deviation respectively, with minimum (min) and maximum (max) values.

In a double-blind, randomized cross-over design, subjects underwent two stepwise incremental exercise tests and two constant load tests (with two phases) on an electrically braked cycle ergometer (Excalibur Sport, Lode, Groningen, The Netherlands). An overview of the design is given in Fig. 1. Each test type was completed under the following conditions: either after oral ingestion of 0.3 g·kg^−1^ BICA, or 4 g sodium chloride (placebo). Both supplements were solved in 0.7 l water. Randomization was performed as simple random allocation; each subject identifier was forwarded to a person who had no further relationship to the conduct of the study, and who performed the randomization blindly using a random draw of numbers. This person also prepared the BICA or placebo drinks, respectively and deposited them in a blank sports bottle in the ergometry lab before entry of subject or testing staff.

**Figure 2:**
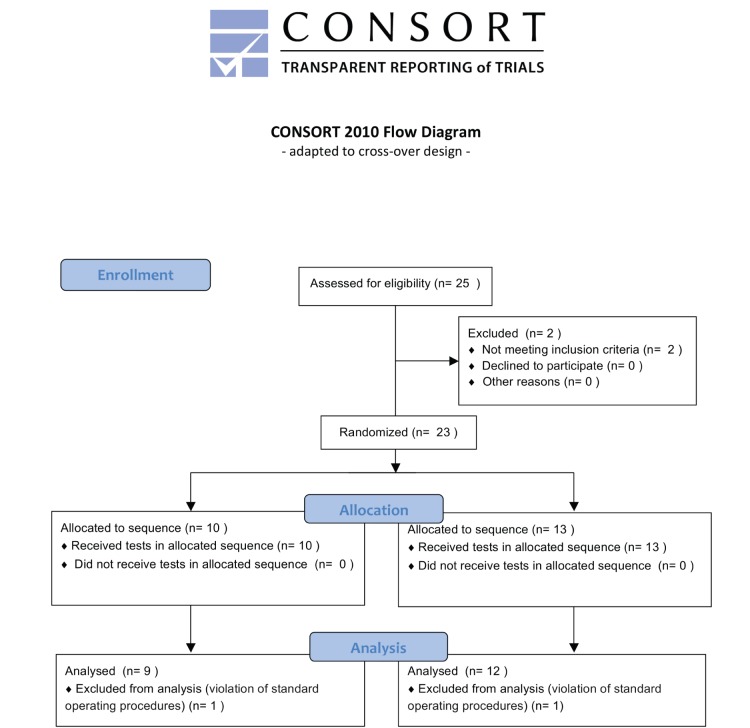
Flow of participants through the study.

Of twenty-five participants included in the study, twenty-one could be included in the analysis (cp. CONSORT flowchart in Fig. 2).

The incremental test with placebo was used to evaluate peak oxygen uptake (VO_2peak_) and the individual anaerobic threshold (IAT). IAT from the placebo test was used to determine the workload applied in the constant load tests.

### Outcome measures

Time to exhaustion in a constant load test, a measure of performance during prolonged, high-intensity exercise to exhaustion, was the main outcome measure in this study. Parameters related to mechanisms for potential improvements in performance (blood lactate [BLa], pH, bicarbonate concentration), as well as parameters of maximum performance in the stepwise incremental exercise test (maximal workload, VO_2peak_), were selected as secondary outcome measures.

### Exercise testing

Test substances were drunk in sips from 2 h to 1 h prior to each test. All trials were performed with clipless pedals and seat position on the cycle ergometer was individualized for each subject. All trials were carried out by the same investigator who was blinded for the administered test solution. Subjects received verbal encouragement throughout the tests. Tests were terminated when subjects signalized maximum exhaustion or were unable to maintain a cadence of 50 rpm (revolutions per minute) for more than 10 s.

#### Incremental exercise test

The stepwise incremental exercise test protocol was as follows: subjects started at an individually chosen workload ranging from 50 to 150 W. Load was increased every 3 min by 50 W until volitional fatigue. During the first trial heart rate was recorded employing 12-lead ECG whereas in the second trial, and the ensuing constant load tests, a telemetric system (Polar S-series, Kempele, Finnland) was used continuously. BLa was determined at rest, at the end of each 3 min step, at exhaustion and 1, 3, 5, 7 and 10 min after cessation of exercise. IAT was determined according to previous methods used by Stegmann et al. [17].

#### Constant load test

The protocol for the constant load tests consisted of 30 min cycling at a resistance equal to 95% IAT followed by exercising at 110% IAT until exhaustion. Prior to each trial subjects completed a standardized warm-up of 2 min at 50 or 100 W. BLa was determined every 5 min and at cessation of exercise.

### Gas exchange measurements

Gas exchange parameters were measured continuously during all exercise tests. Subjects breathed through a Hans-Rudolph-facemask, which was attached to a “Triple V” digital flow meter measuring ventilation volume. Expired gases were continuously collected and analyzed with the MetaMax II metabolic test system (Cortex Biophysik, Leipzig, Germany; mixing chamber; sampling frequency 10 s). Before each test the flow meter was calibrated using a 3-L syringe and gas sensors were gauged by commercial calibration gas (2-point calibration) according to the manufactureŕs recommendations. Test-specific maximal respiratory parameters were calculated as the mean of the 3 highest values during exercise.

### Blood sampling and analysis

Arterialized capillary blood was obtained from the hyperemized earlobe and analyzed for BLa (enzymatic-amperometric method, Greiner, Flacht, Germany) and blood gas parameters (ABL5 blood gas analyzer, Radiometer, Willich, Germany). Measured blood gas parameters were pH, pO_2_ and pCO_2_ from which bicarbonate concentration, oxygen saturation and base excess (BE) were calculated [18]. The blood gas analyzer was calibrated prior to each test. Blood gas parameters were measured 2 h and immediately before testing (pre and post drink), every 5 min during the constant load tests, at exhaustion and 2 min post assuming that this represents the time point of largest extracellular acidosis [19].

### Statistical analysis

Sample size was estimated with the program G*Power (version 3.1.5) using the following parameters: effect size to be detected 0.6, alpha error probability 0.05, 1-beta error probability 0.8; 1-tailed testing. The calculated sample size of 19 was rounded up to 20. To allow for potential drop-outs 25 subjects were included in the study.

Because normal distribution was present for all dependent variables (Shapiro-Wilk-W-test), parametric tests were applied and data are presented as means ± standard deviation. Differences between the BICA and placebo conditions were tested for significance using one-factorial analysis of variance (ANOVA; factor: condition). Courses of BLa and blood gas parameters during constant load tests were compared using two-factorial ANOVA for repeated measures (factor one: condition, factor two: time of measurement). When the difference between means was significant, post-hoc comparisons were performed with the Scheffé test. The significance level for the alpha error was set at p<0.05.

## Results

### Performance

Time to exhaustion during the constant load tests was significantly increased (p<0.05) under BICA (49.5±11.5 min) compared to placebo (45.0±9.5 min). Maximal workload in the stepwise incremental tests (BICA: 341±66 W; placebo: 339±67 W) and workload at IAT (BICA: 234±5.5 W; placebo 233±5.7 W) did not differ between conditions. In this context, exercise lactate curves showed a parallel shift along the y-axis (factor condition) but not along the x-axis (interaction).

### Acid-base-status

**Figure 3:**
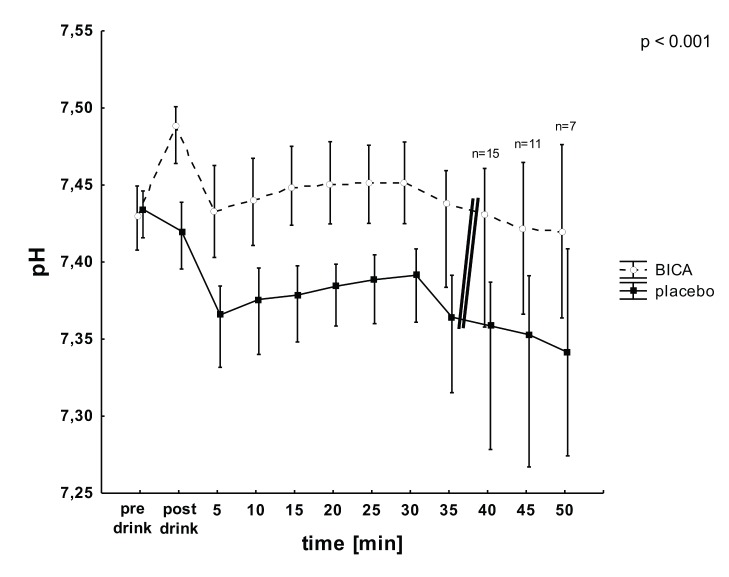
pH-levels before (pre drink), after ingestion (post drink) and during constant load tests (mean ± SD) for the BICA (open circles) and placebo (closed squares) trials. Double bar = decrease in number of exercising subjects (n<21). p = significant interaction between condition and time.

All baseline blood gas parameters were similar and within normal ranges. In both test types BICA was associated with extracellular metabolic alkalosis 1 h after ingestion at rest indicated by increased pH, bicarbonate concentration and BE. Changes in acid-base-status during the constant load tests and incremental tests are shown in table 1 and 2, respectively. Throughout the constant load tests pH remained elevated in the BICA-treatment as compared to placebo condition (Fig. 3). Under both conditions a pH nadir was reached 2 min after exercise. While the lowest pH with BICA was similar to resting-value just before placebo-trial, the lowest pH with placebo was slightly within acidic range.

**Table 1:** Blood gas parameters during constant load tests.

	BICA (n = 21)	Placebo (n = 21)	p-value
**pH**			
Post drink	7.48±0.02	7.42±0.02	**<0.001**
30 min	7.45±0.03	7.38±0.03	**<0.001**
2 min post-exercise	7.39±0.05	7.32±0.07	**<0.001**
**Bicarbonate** [mmol*l^−1^]			
Post drink	31.3±1.4	24.9±1.5	**<0.001**
30 min	27.6±1.7	21.4±2.0	**<0.001**
2 min post-exercise	20.2±4.0	16.4±2.9	**<0.001**
**Base Excess** [mmol*l^−1^]			
Post drink	7.4±1.2	0.7±1.5	**<0.001**
30 min	3.1±1.6	−2.6±1.7	**<0.001**
2 min post-exercise	−3.2±3.7	−8.6±3.8	**<0.001**
**pCO_2_** [mmHg]			
Post drink	42.2±2.3	39.2±2.4	**<0.001**
30 min	38.8±3.2	36.5±3.9	**<0.001**
2 min post-exercise	33.8±4.9	32.5±3.4	0.33
**pO_2_** [mmHg]			
Post drink	81.8±8.1	89.0±7.6	**<0.01**
30 min	71.2±6.1	75.7±6.9	**<0.01**
2 min post-exercise	83.4±6.5	88.0±6.2	**<0.01**
**Oxygen saturation** [%]			
Post drink	96.4±1.3	96.7±0.8	0.30
30 min	94.5±1.1	94.6±1.5	0.72
2 min post-exercise	96.0±1.4	95.7±1.4	0.35

pH, bicarbonate concentration, base excess, partial pressure of carbon dioxide (pCO_2_), partial pressure of oxygen (pO_2_) and oxygen saturation were determined at rest (post drink), after 30 minutes cycling and 2 minutes post-exercise. Values are means ± SD.

Significance testing: one-factorial repeated measures analysis of variance (factor: condition; post-hoc: Scheffé test).

**Table 2:** Blood gas parameters before and after incremental tests.

	BICA (n = 21)	Placebo (n = 21)	p-value
**pH**			
Pre drink	7.43±0.02	7.44±0.02	0.14
Post drink	7.48±0.02	7.42±0.01	**<0.001**
2 min post-exercise	7.24±0.07	7.18±0.07	**<0.001**
**Bicarbonate** [mmol*l^−1^]			
Pre drink	25.0±1.5	24.9±1.4	0.41
Post drink	31.1±1.5	24.1±1.9	**<0.001**
2 min post-exercise	13.6±2.4	11.4±2.2	**<0.001**
**Base Excess** [mmol*l^−1^]			
Pre drink	1.2±1.2	1.4±1.4	0.44
Post drink	7.3±1.3	0.3±1.5	**<0.001**
2 min post-exercise	−12.5±3.2	−16.2±3.6	**<0.001**
**pCO_2_** [mmHg]			
Pre drink	38.6±2.7	37.6±2.5	0.13
Post drink	42.0±2.7	38.3±2.9	**0.03**
2 min post-exercise	32.8±4.6	31.2±3.1	0.46
**pO_2_** [mmHg]			
Pre drink	83.3±6.9	83.8±5.9	0.14
Post drink	80.2±7.8	85.6±5.2	**<0.001**
2 min post-exercise	89.7±4.9	95.1±5.1	**<0.001**
**Oxygen saturation** [%]			
Pre drink	96.2±0.8	96.4±0.6	0.29
Post drink	96.2±1.2	96.6±0.8	0.34
2 min post-exercise	95.7±1.2	95.5±1.1	0.56

pH, bicarbonate concentration, base excess, partial pressure of carbon dioxide (pCO_2_), partial pressure of oxygen (pO_2_) and oxygen saturation were determined at rest (pre and post drink) and 2 minutes post-exercise. Values are means ± SD.

Significance testing: one-factorial repeated measures analysis of variance (factor: condition; post-hoc: Scheffé test).

In the BICA-trial bicarbonate concentration remained higher compared to pre-ingestion value after 30 min cycling but was lower again at exhaustion. Compared to placebo, post-ingestion bicarbonate-levels remained higher with BICA throughout the constant load tests (p<0.001). Similarly BE remained increased with BICA after 30 min and dropped considerably below its initial value when the constant load test was finished. The pCO_2_-values were continuously elevated after ingestion of BICA. The time course of pO_2_ did not show an interaction effect between the condition and time factors. No difference was observed for oxygen saturation throughout the constant load tests.

During the incremental tests the increase of pH, BE and bicarbonate concentration after ingestion of BICA at rest was maintained until 2 minutes post-exercise, when largest acidosis occured. In addition, pO_2_ was decreased with BICA just after cessation of exercise, while oxygen saturation remained unchanged throughout the test.

### Blood lactate concentration

**Figure 4:**
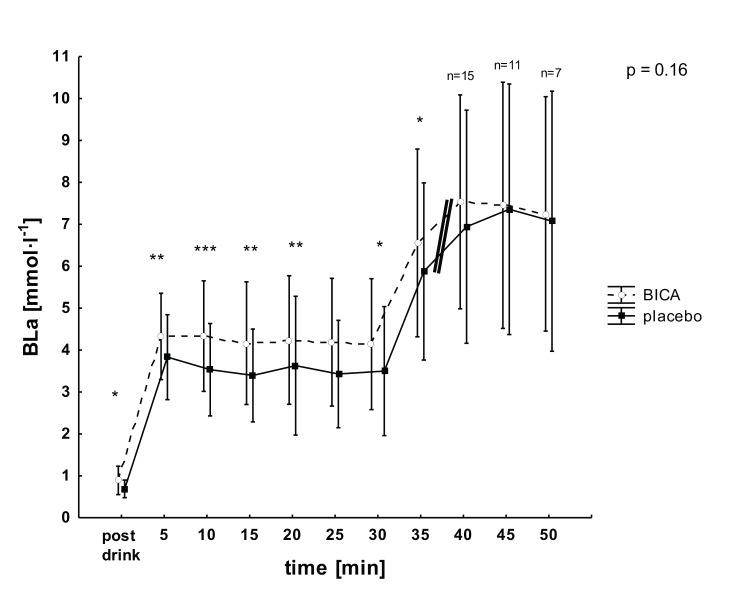
Blood lactate (BLa) concentrations after ingestion (post drink) and during constant load tests (mean ± SD) for the BICA (open circles) and placebo (closed squares) trials. Double bar = decrease in number of exercising subjects (n<21). p = interaction between condition and time. * significant effect of condition, p<0.05; ** significant effect of condition, p<0.01; *** significant effect of condition, p<0.001.

Baseline BLa with BICA was elevated compared to placebo for both test types (stepwise incremental tests 0.9±0.3 vs. 0.7±0.2 mmol·l^−1^, p = 0.04; constant load tests 0.8±0.3 vs. 0.7±0.2 mmol·l^−1^, p = 0.02). Time courses of BLa during the constant load tests did not differ between conditions as indicated by the absence of significant interaction between the condition and time factors. However, a significant global effect was present for the condition factor and post-hoc tests confirmed higher BICA concentrations for all time points except for the 25-minute value (Fig. 4). Moreover, BLa at exhaustion was elevated with BICA in the constant load tests (p<0.01) as well as in the incremental tests (p<0.01).

### Gas exchange measurements

The test specific highest oxygen uptake during the constant load tests did not differ significantly between conditions (BICA: 58.6±8.6 ml·kg^−1^·min^−1^, placebo: 57.6±8.8 ml·kg^−1^·min^−1^). However, during stepwise incremental tests VO_2peak_ was slightly decreased (p<0.05) with BICA (64.0±8.6 ml·kg^−1^·min^−1^) compared to placebo (67.3±9.8 ml·kg^−1^·min^−1^). Respiratory exchange ratio at exhaustion did not differ significantly for the constant load tests but was increased with BICA for the stepwise incremental test (p<0.001).

### Heart rate

The course of exercise heart rate as well as maximum heart rate did not differ significantly between conditions for both test types.

## Discussion

The present study demonstrates an ergogenic effect of oral BICA ingestion for prolonged cycling slightly above the anaerobic threshold. Specifically the intake of 0.3 g·kg^−1^ BICA was associated with an increase in time to exhaustion at 110% IAT. No subject reported gastrointestinal discomfort or felt impaired in performance. While in a recent meta-analysis evidence was shown in support of an ergogenic effect of BICA for sprint-type exercise [11], only few studies with small samples examined the effect of BICA on prolonged cycling performance [12]–[14].

In the study of McNaughton et al. [12] cyclists performed on average 14% more work with BICA compared to placebo in a 60-min time trial [12]. In contrast, Stephens et al. [13] reported a significant muscle alkalosis but no difference in performance between BICA and placebo in a trial consisting of 30 min cycling at 77% VO_2peak_ followed by completing as much work as possible in another 30 min (mean intensity equivalent to 80% VO_2peak_) [13]. Our subjects’ characteristics were similar to Stephens et al. [13], whereas exercise intensity was higher after 30 min in our study (on average 85% VO_2peak_ equivalent to 110% IAT). It is conceivable that subjects in the study of Stephens et al. [13] worked just below, or at IAT, and thus did not benefit from the increased buffering capacity. Nevertheless, McNaughton et al. [12] assumed that their participants did not exercise for a long period of time above the IAT though BICA ingestion showed an ergogenic effect. Mitchell et al. [14] observed no difference in cycling time to exhaustion at exercise intensity similar to that of Stephens et al. [13] (∼80% VO_2max_) when comparing BICA to sodium chloride. In these studies [13], [14] exercise intensity above the IAT remains unclear, since exercising at given percentages of VO_2max_ is associated with high variability of BLa responses [20]. During more anaerobic prolonged intermittent cycling, BICA improves sprint performance, although this protocol may be more representative of team sports [21].

Apart from cycling, two studies have previously examined the effect of BICA on running endurance performance [15], [16]. George et al. [15] reported a numerical increase of 17% in time to exhaustion, which failed to reach statistically significance based on the small sample size (n = 7). Likewise, Potteiger et al. [16] observed no statistically significant difference between BICA and placebo in seven runners using a test protocol similar to our study. Statistical power is not specified in either of these cases.

The significant elevation of blood pH (0.05±0.02) and bicarbonate concentration (6.1±1.6 mmol·l^−1^) after ingestion of BICA was of similar magnitude compared to previous investigations [2], [21]. Both values remained unchanged after placebo and differences between conditions were maintained throughout the constant load tests. Moreover, the exercise-induced decline in pH from preingestion values to exhaustion was smaller with BICA (0.03±0.05) compared to placebo (0.11±0.06). 2 min after cessation of constant load tests only a slight acidosis occurred with placebo, whereas pH remained within standard range with BICA. We therefore agree with others that pH does not seem to be the limiting factor during such endurance performance [13], [14]. Likewise, the ergogenic effect of BICA does not seem to be limited to – or ensured by - exercise protocols leading to severe lactic acidosis. This is further substantiated by the results of stepwise incremental test, where no difference in performance became apparent between BICA and placebo despite a severe acidosis.

In agreement with earlier reports BLa-levels remained higher during exercise after BICA-induced metabolic alkalosis in both test types [2], [4], [13]. This may be due to a higher efflux of lactate from the working muscle resulting from an increased H^+^-concentration gradient across the sarcolemma due to extracellular alkalosis [3], [9]. Lactate removal from muscle is mediated by membrane associated lactate/H^+^-cotransporters (monocarboxylate-transporters), which are sensitive to the concentration gradients of both ions [22]. However, H^+^ seems to be the main effector with induced alkalosis [9]. In addition, it has been reported that imposed metabolic alkalosis results in acceleration of glycogenolysis and glycolysis leads to a mismatch in pyruvate production and oxidation and subsequent increase in BLa production [9]. Therefore an elevated glycolytic flux is likely to be responsible for the elevated BLa after BICA ingestion.

Increased glycolysis and glycogenolysis [9] may lead to accelarated consumption of glycogen stores and premature exhaustion in the case of long term endurance exercise. However, the relevance of this potential side effect remains to be investigated.

In contrast to constant load tests, maximal and submaximal performance were unchanged during the stepwise incremental cycle test in our subjects. Probably, exercise time at maximal intensity was not long enough to profit from increased buffering capacity. Moreover, the absence of an ergogenic effect with BICA during stepwise incremental tests could also be explained by a limitation of aerobic metabolism. Despite similar maximum heart rate, VO_2peak_ was significantly lower with BICA compared to placebo. This may be due to the well-known left shift of the oxyhemoglobin dissociation curve during alkalosis [23], evidenced by lowered pO_2_ with BICA and unchanged oxygen saturation between conditions. It is possible that this impairment of oxygen respiration was compensated for by the aforementioned increase in glycolytic flux and concomitant adenosine triphosphate production. In contrast, oxygen uptake was unaltered during the (submaximal) constant load test. Therefore, the accelerated glycolytic flux and consecutive performance enhancement came into effect.

A comprehensive assessment of the physiological consequences of BICA-induced metabolic alkalosis is beyond the scope of the present study. However, it seems clear that increased buffering capacity and its influence on glycolytic flux are not the only factors with a potential influence on physical performance. While the classical theory of lactate production causing muscular fatigue is strongly challenged [24], [25], it has been shown that lactate triggers positive effects on muscle performance resulting in preservation of muscle excitability [24]. In addition, lactate as a weak base is able to buffer H^+^-ions. Thus, the higher lactate level by itself may have contributed to performance enhancement with BICA. Moreover, BICA reduces the extracellular accumulation of potassium (K^+^) after high-intensity exercise [26], [27], thereby potentially counteracting K^+^-induced depression of muscle force [8].

### Limitations

BICA has the potential to improve performance during prolonged high-intensity cycling. However, several aspects have to be considered when practical application of BICA is envisaged. First, although prolonged high-intensity exercise is representative of road-cycling competitions, the variation in load will be higher than during a constant-load exercise test. This is particularly true for pack-based competitions, but holds true even for time-trials due to individual pacing strategies. Therefore, the effectiveness of BICA under race conditions warrants further study. Second, time-trial performance is generally regarded as having a lower coefficient of variation indicating better reproducibility as compared to TTE [28]. However, as a significant effect of BICA could be demonstrated, this potential decrease in standardized effect sizes seems unlikely to have affected our results.

With respect to maximum performance in the incremental exercise test, which was mainly conducted to allow for the determination of IAT, it has to be kept in mind that stepwise tests are less optimized for determination of maximal parameters of performance than ramp-shaped tests [29].

## Conclusion

The results of the present study have shown that the ergogenic potential of BICA, which has been conclusively demonstrated in previous work for sprint-type exercise, may also be seen with prolonged exercise. However, the interindividual variability in the difference between tests under BICA or placebo conditions was high. Moreover, the transferability of these results to real-world race conditions remains to be demonstrated.

## Supporting Information

S1 Checklist **CONSORT Checklist.**
(PDF)

S1 Data **Tabular presentation of all relevant data.**
(PDF)

S1 Protocol **Study protocol.** The original study protocol has been carefully translated into English language.
(PDF)

S2 Protocol **Study protocol in original language (German).**
(PDF)
