# Supplementary material for: Effects of Sodium Bicarbonate on High-Intensity Endurance Performance in Cyclists: A Double-Blind, Randomized Cross-Over Trial
Source: PLoS One. 2014 Dec 10;9(12):e114729. doi: 10.1371/journal.pone.0114729 (PMC4262454; doi:10.1371/journal.pone.0114729)
Supplement: S1 Data — Tabular presentation of all relevant data. (PDF) [file pone.0114729.s002.pdf]

Baseline characteristics

n	gender<br>m=1, f=2	type of sport<br>rb =1, mb=2, tri=3	age<br>[y]	height<br>[cm]	weight<br>[kg]	BMI<br>[kg/m^2]	VO_{2peak}<br>[$ml \cdot min^{-1} \cdot kg^{-1}$]
1	1	2	28	188	78	20.1	76.6
2	1	1	27	183	69	20.7	77.0
3	1	3	21	178	69	21.8	70.3
4	2	3	24	171	50	17.1	63.5
5	1	1	23	172	63	21.3	75.2
6	2	1	19	173	59	19.7	62.6
7	1	1	27	186	76	22.0	78.6
8	1	2	20	178	76	23.9	71.3
9	1	2	18	180	71	21.9	84.6
10	1	2	18	183	72	21.5	73.8
11	1	1	24	179	70	21.8	57.9
12	1	2	19	178	72	22.7	71.4
13	2	1	44	182	74	22.3	50.1
14	1	2	20	174	65	21.5	63.0
15	1	2	18	191	85	23.4	71.3
16	1	1	25	181	69	21.1	64.6
17	1	2	21	172	69	23.3	71.8
18	1	2	19	179	64	20.0	66.3
19	1	3	22	178	71	22.4	65.0
20	2	1	47	168	51	18.1	47.4
21	2	1	28	159	52	20.6	50.5

m= male; f= female; rb = racing bike; mb= mountain bike; tri= triathlon

incremental exercise test (placebo)

n	pH pre	pH post	pH 2 min p	BIC pre [mmol·l^{-1}]	BIC post [mmol·l^{-1}]	BIC 2 min p [mmol·l^{-1}]	BE pre [mmol·l^{-1}]	BE post [mmol·l^{-1}]	BE 2 min p [mmol·l^{-1}]
1	7.43	7.42	7.18	24	25	11	1	1	-17
2	7.41	7.41	7.07	25	24	9	1	0	-21
3	7.44	7.43	7.21	26	26	13	3	2	-14
4	7.45	7.43	7.26	22	22	10	-1	-1	-16
5	7.42	7.40	7.11	25	25	10	1	1	-19
6	7.42	7.42	7.03	22	22	7	-1	-1	-23
7	7.44	7.42	7.12	25	24	10	2	1	-19
8	7.45	7.41	7.23	25	24	14	2	0	-12
9	7.44	7.41	7.20	26	25	12	2	1	-16
10	7.45	7.43	7.18	26	25	13	3	1	-15
11	7.42	7.42	7.14	25	26	10	1	2	-18
12	7.41	7.42	7.15	24	24	9	0	0	-19
13	7.44	7.41	7.27	24	18	12	1	-5	-13
14	7.42	7.41	7.20	26	25	12	2	0	-15
15	7.44	7.43	7.23	26	26	14	3	2	-12
16	7.43	7.42	7.30	26	24	16	2	0	-9
17	7.44	7.41	7.10	26	25	9	2	1	-20
18	7.50	7.43	7.11	27	26	10	4	2	-20
19	7.43	7.41	7.19	26	25	11	2	1	-16
20	7.44	7.43	7.28	23	23	14	0	0	-12
21	7.42	7.42	7.21	23	23	13	-1	-1	-14

pre = pre drink; post = post drink; 2 min p= 2 min post exercise; BIC = bicarbonate concentration; BE = base excess

incremental exercise test (placebo)

n	pCO_2 pre [mmHg]	pCO_2 post [mmHg]	pCO_2 2 min p [mmHg]	pO_2 pre [mmHg]	pO_2 post [mmHg]	pO_2 2 min p [mmHg]	SO_2 pre [%]	SO_2 post [%]	SO_2 2min p [%]
1	37	39	29	86	90	95	97	97	96
2	39	40	32	90	94	89	97	97	93
3	40	40	33	83	85	96	96	97	96
4	32	34	23	94	95	108	97	97	97
5	39	41	33	81	88	93	96	99	94
6	35	36	27	89	84	98	97	96	94
7	38	38	33	89	83	95	97	96	95
8	37	39	35	84	85	88	97	96	95
9	39	40	31	79	87	103	96	97	97
10	39	39	35	75	80	95	95	96	96
11	39	40	32	78	79	97	96	96	95
12	38	38	28	95	90	95	97	97	95
13	36	29	28	86	75	91	97	95	96
14	41	40	30	86	80	103	96	96	97
15	39	41	36	77	78	88	96	96	95
16	39	38	33	79	91	90	96	97	96
17	39	40	31	84	86	97	96	96	95
18	35	40	31	76	85	91	96	97	94
19	40	41	31	79	85	96	96	96	96
20	32	36	30	79	89	96	96	97	97
21	36	36	34	91	89	93	97	97	96

pre = pre drink; post = post drink; 2 min p= 2 min post exercise; pCO_2 = partial pressure of carbon dioxide; pO_2 = partial pressure of oxygen; SO_2 = oxygen saturation

incremental exercise test (placebo)

n	HR rest [bpm]	HR max [bpm]	P max [W]	P max [W·kg^{-1}]	VO_{2peak} [ml·min^{-1}]	VO_{2peak} [ml·min^{-1}·kg^{-1}]	RER	Lac rest [mmol·l^{-1}]	Lac max [mmol·l^{-1}]	IAT [W]	IAT [W·kg^{-1}]
1	75	193	450	5.9	5825	76.6	1.20	0.7	12.9	351	4.5
2	58	200	408	5.8	5533	77.0	1.12	0.8	15.0	292	4.2
3	77	186	342	5.0	4781	70.3	1.13	0.7	13.6	230	3.3
4	66	184	233	4.7	3175	63.5	1.18	1.0	10.5	150	3.0
5	51	188	325	5.2	4924	75.2	1.24	0.8	14.3	216	3.4
6	67	185	283	4.8	3694	62.6	1.19	0.8	15.0	180	3.1
7	60	185	383	5.1	5891	78.6	1.19	0.7	13.4	284	3.7
8	88	202	367	5.0	5608	71.3	1.13	0.6	8.4	259	3.4
9	88	202	417	5.9	6006	84.6	1.02	0.9	10.5	299	4.2
10	64	186	400	5.6	5312	73.8	1.04	1.1	11.0	290	4.0
11	69	179	333	4.8	4025	57.9	1.16	0.6	14.0	228	3.3
12	51	195	400	5.4	4844	71.4	1.18	0.8	13.0	263	3.7
13	72	162	292	4.0	3551	50.1	1.17	0.6	9.8	195	2.6
14	57	184	300	4.7	4033	63.0	1.21	0.5	12.5	200	3.1
15	86	212	392	4.6	6062	71.3	1.03	0.5	9.6	190	2.2
16	83	199	325	4.7	4460	64.6	1.16	0.5	8.1	240	3.5
17	66	189	375	5.4	4954	71.8	1.11	0.7	14.0	268	3.9
18	98	200	358	5.6	4244	66.3	1.30	0.7	14.5	262	4.1
19	76	195	325	4.6	4618	65.0	1.16	0.8	12.0	232	3.3
20	53	170	208	4.1	2303	47.4	1.22	0.6	9.0	138	2.7
21	76	201	208	4.0	2628	50.5	1.32	0.7	9.5	133	2.6

HR rest = heart rate at rest; HR max = maximum heart rate; P max= maximum performance; RER= respiratory exchange ratio; Lac rest= lactate concentration at rest; Lac max= lactate concentration at exhaustion; IAT= individual anaerobic threshold

incremental exercise test (BICA)

n	pH pre	pH post	pH 2 min p	BIC pre [mmol·l^{-1}]	BIC post [mmol·l^{-1}]	BIC 2 min p [mmol·l^{-1}]	BE pre [mmol·l^{-1}]	BE post [mmol·l^{-1}]	BE 2 min p [mmol·l^{-1}]
1	7.41	7.48	7.30	25	31	14	1	7	-10
2	7.43	7.49	7.20	26	32	12	2	8	-15
3	7.43	7.46	7.24	26	31	15	2	6	-12
4	7.45	7.49	7.38	21	29	9	-2	5	-14
5	7.40	7.44	7.17	25	32	12	1	7	-16
6	7.43	7.47	7.16	24	28	10	1	5	-18
7	7.42	7.50	7.17	26	30	12	2	7	-15
8	7.42	7.47	7.30	25	32	16	1	8	-9
9	7.43	7.50	7.24	25	31	14	1	8	-12
10	7.42	7.49	7.23	28	34	16	3	10	-11
11	7.44	7.50	7.19	26	33	13	2	9	-15
12	7.44	7.49	7.20	25	31	12	2	7	-15
13	7.46	7.50	7.40	24	32	18	1	8	-5
14	7.42	7.46	7.25	26	32	15	2	8	-11
15	7.44	7.49	7.21	26	32	14	2	8	-13
16	7.39	7.48	7.29	24	31	17	0	7	-9
17	7.40	7.46	7.15	25	31	11	0	7	-17
18	7.45	7.49	7.18	27	33	12	3	9	-15
19	7.42	7.47	7.22	25	30	12	1	6	-12
20	7.44	7.48	7.34	23	29	17	0	6	-8
21	7.44	7.52	7.26	24	30	15	0	7	-11

pre = pre drink; post = post drink; 2 min p= 2 min post exercise; BIC = bicarbonate concentration; BE = base excess

incremental exercise test (BICA)

n	pCO_2 pre [mmHg]	pCO_2 post [mmHg]	pCO_2 2 min p [mmHg]	pO_2 pre [mmHg]	pO_2 post [mmHg]	pO_2 2 min p [mmHg]	SO_2 pre [%]	SO_2 post [%]	SO_2 2min p [%]	interval [d]
1	40	42	30	97	87	92	97	97	97	7
2	39	42	33	79	84	87	96	97	95	7
3	40	44	36	75	76	86	95	96	95	8
4	31	38	16	101	88	96	98	97	99	6
5	41	49	34	78	62	86	95	92	94	6
6	37	39	28	80	77	96	96	96	97	7
7	40	39	35	80	92	87	96	98	94	7
8	39	44	35	82	74	84	96	95	95	16
9	39	41	34	92	85	90	97	97	96	10
10	43	45	39	75	84	88	95	97	95	7
11	39	43	34	80	74	90	96	96	95	7
12	38	41	32	82	70	79	96	95	95	7
13	34	41	30	83	75	85	97	96	96	7
14	41	45	35	90	78	96	97	96	96	7
15	38	43	37	85	76	94	97	96	96	7
16	40	41	35	75	86	86	95	97	95	5
17	41	43	33	84	88	91	96	97	95	4
18	39	44	35	88	76	86	97	96	94	6
19	40	41	30	83	92	96	96	97	96	8
20	35	40	32	78	74	93	96	95	97	7
21	36	38	35	83	86	95	96	97	96	7

pre = pre drink; post = post drink; 2 min p= 2 min post exercise; pCO_2 = partial pressure of carbon dioxide;
pO_2 = partial pressure of oxygen; SO_2 = oxygen saturation; interval= days between incremental exercise tests

incremental exercise test (BICA)

n	HR rest [bpm]	HR max [bpm]	P max [W]	P max [W·kg^{-1}]	VO_{2peak} [ml·min^{-1}]	VO_{2peak} [ml·min^{-1}·kg^{-1}]	RER	Lac rest [mmol·l^{-1}]	Lac max [mmol·l^{-1}]	IAT [W]	IAT [W·kg^{-1}]
1	65	188	417	5.4	5763	72.8	1.15	0.5	13.8	322	4.1
2	76	202	417	6.0	5161	73.7	1.27	0.9	18.1	280	4.1
3	54	187	350	5.1	4527	65.6	1.29	0.6	13.0	222	3.2
4	46	190	233	4.7	2799	60.9	1.23	0.9	13.8	136	2.7
5	60	189	325	5.2	4352	69.1	1.33	0.8	14.8	207	3.3
6	62	185	289	4.9	3966	72.2	1.21	1.1	15.3	201	3.4
7	79	184	383	5.0	5599	73.7	1.28	1.3	15.8	280	3.7
8	72	207	367	4.8	5420	70.8	1.09	0.6	10.2	273	3.6
9	83	201	417	6.0	5247	76.0	1.16	0.6	13.2	279	3.9
10	75	191	408	5.6	4958	67.9	1.23	1.2	14.5	290	4.0
11	68	179	342	5.0	4040	58.6	1.27	1.0	16.7	242	3.5
12	84	192	400	5.6	5006	70.5	1.21	1.1	14.6	286	4.0
13	59	165	288	4.0	3504	49.3	1.25	0.9	9.9	190	2.6
14	63	183	300	4.6	4056	60.4	1.23	0.6	13.9	198	3.0
15	67	208	400	4.7	6013	67.7	1.06	0.5	14.5	271	3.2
16	83	193	328	4.7	4383	60.6	1.24	0.6	10.9	240	3.5
17	63	191	383	5.5	4848	69.3	1.18	0.9	17.0	250	3.6
18	81	198	358	5.6	4352	68.0	1.20	0.7	18.4	253	4.0
19	74	200	342	4.8	4279	60.7	1.32	0.8	16.4	223	3.1
20	64	174	213	4.2	2393	45.9	1.22	0.6	10.1	132	2.6
21	75	204	211	4.0	2595	51.4	1.49	1.2	12.7	128	2.5

HR rest = heart rate at rest; HR max = maximum heart rate; P max= maximum performance; RER= respiratory exchange ratio;
Lac rest= lactate concentration at rest; Lac max= lactate concentration at exhaustion; IAT= individual anaerobic threshold

constant load test (placebo)

n	ph pre	ph post	ph 5 min	ph 10 min	ph 15 min	ph 20 min	ph 25 min	ph 30 min	ph 35 min	ph 40 min	ph 45 min	ph 50 min	pH 2 min p
										n=15	n=11	n=7	
1	7.41	7.41	7.38	7.41	7.40	7.39	7.39	7.39	7.39				7.39
2	7.45	7.47	7.36	7.37	7.37	7.36	7.34	7.34	7.29	7.27	7.23		7.23
3	7.43	7.44	7.39	7.39	7.38	7.38	7.40	7.40	7.38	7.36			7.36
4	7.44	7.44	7.39	7.41	7.41	7.42	7.43	7.42	7.41	7.39	7.40	7.39	7.39
5	7.41	7.41	7.34	7.34	7.34	7.34	7.36	7.35	7.32	7.30	7.29	7.28	7.23
6	7.43	7.41	7.37	7.35	7.36	7.37	7.36	7.37	7.32	7.31	7.29	7.24	7.24
7	7.42	7.43	7.34	7.35	7.35	7.37	7.37	7.38	7.35	7.30	7.25		7.25
8	7.43	7.42	7.38	7.39	7.38	7.38	7.38	7.39	7.38	7.38	7.38	7.34	7.34
9	7.44	7.39	7.37	7.36	7.36	7.37	7.36	7.37	7.35	7.32	7.30		7.30
10	7.41	7.42	7.32	7.34	7.37	7.37	7.38	7.37	7.37	7.36			7.35
11	7.42	7.41	7.39	7.38	7.38	7.38	7.39	7.40	7.35				7.35
12	7.43	7.40	7.33	7.35	7.34	7.36	7.36	7.36	7.34	7.33	7.32	7.32	7.29
13	7.46	7.46	7.41	7.43	7.44	7.43	7.43	7.45	7.40	7.41	7.41	7.43	7.44
14	7.41	7.40	7.33	7.35	7.36	7.37	7.38	7.37	7.35				7.32
15	7.44	7.41	7.37	7.40	7.39	7.39	7.39	7.39	7.39				7.39
16	7.46	7.41	7.35	7.36	7.37	7.37	7.39	7.39	7.37				7.37
17	7.42	7.39	7.37	7.38	7.39	7.39	7.39	7.37	7.29				7.29
18	7.43	7.43	7.32	7.33	7.35	7.37	7.36	7.35	7.27	7.21			7.21
19	7.44	7.42	7.33	7.34	7.34	7.36	7.39	7.39	7.34	7.29			7.29
20	7.43	7.39	7.34	7.34	7.37	7.39	7.38	7.41	7.38	7.37	7.37		7.37
21	7.44	7.40	7.34	7.36	7.38	7.39	7.40	7.39	7.38	7.39	7.38	7.39	7.40

pre = pre drink; post = post drink; 2 min p = 2 min post exercise

constant load test (placebo)

n	BIC post [mmol·l^{-1}]	BIC 30 min [mmol·l^{-1}]	BIC 2 min p [mmol·l^{-1}]	BE post [mmol·l^{-1}]	BE 30 min [mmol·l^{-1}]	BE 2 min p [mmol·l^{-1}]	pCO_2 post [mmol·l^{-1}]	pCO_2 30 min [mmol·l^{-1}]	pCO_2 2 min p [mmol·l^{-1}]	pO_2 post [mmol·l^{-1}]	$pO_2$30 min [mmol·l^{-1}]	pO_2 2 min p [mmol·l^{-1}]
1	25	19	15	1	-4	-10	40	33	33	95	82	99
2	27	23	14	3	-2	-12	37	43	33	91	66	89
3	26	23	20	3	-1	-4	39	38	37	85	78	78
4	21	19	16	-2	-4	-8	32	30	26	78	88	91
5	25	20	12	0	-4	-15	39	36	29	85	69	72
6	24	19	12	0	-5	-14	39	34	28	96	76	89
7	26	21	14	2	-3	-12	40	37	34	79	75	81
8	26	25	18	2	0	-7	41	41	34	76	71	89
9	24	21	16	-1	-3	-9	40	36	33	90	76	89
10	26	25	20	2	0	-4	41	45	37	80	63	86
11	27	22	20	2	-2	-5	43	37	36	80	66	92
12	24	22	15	0	-3	-11	40	40	32	90	75	92
13	25	22	17	2	-1	-5	36	32	27	95	80	84
14	25	21	16	0	-3	-9	40	37	34	93	91	93
15	24	22	21	0	-2	-4	39	37	38	95	78	97
16	25	22	18	0	-2	-6	40	37	33	99	78	88
17	24	18	14	0	-6	-11	41	31	30	99	71	84
18	27	19	13	3	-5	-15	42	35	33	93	78	86
19	24	22	15	0	-2	-10	37	36	31	97	73	90
20	22	20	16	-2	-3	-8	37	32	28	79	82	94
21	25	24	22	0	0	-2	41	40	36	93	73	85

post = post drink; 2 min p= 2 min post exercise; BIC = bicarbonate concentration; BE = base excess;
pCO_2 = partial pressure of carbon dioxide; pO_2 = partial pressure of oxygen

constant load test (placebo)

n	SO_2 post [mmol·l^{-1}]	SO_2 30 min [mmol·l^{-1}]	SO_2 2 min [mmol·l^{-1}]	interval [d]	interval tt [d]	95% IAT [W]	110% IAT [W]	TTE [min]	VO_2 top [ml·min^{-1}]	VO_2 top [ml·min^{-1}·kg^{-1}]	RER
1	97	96	97	7	14	333	386	33.5	5292	67.9	1.04
2	97	92	95	6	32	277	321	44	4857	68.9	1.01
3	97	95	95	7	15	219	253	38	3832	55.5	0.99
4	96	97	97	6	13	143	165	50	2774	56.5	0.99
5	96	93	91	7	7	205	238	58	3905	62.0	1.02
6	97	95	96	7	7	171	198	48	3225	56.4	1.04
7	96	95	94	7	15	270	312	46	4611	60.7	1.06
8	95	94	96	9	13	246	285	52	4754	62.1	1.01
9	97	95	96	6	18	284	329	45	4972	70.0	1.03
10	96	91	96	8	6	276	319	48	4685	65.1	0.96
11	96	93	97	6	14	217	251	37	3627	51.1	1.00
12	97	94	96	14	7	250	289	60	4586	63.7	1.04
13	98	96	97	9	11	185	215	60	3177	42.6	1.03
14	97	97	96	7	8	190	220	40	3506	56.8	1.03
15	97	95	97	15	7	276	319	33	4513	52.5	0.97
16	98	95	96	5	32	228	264	35	3749	54.3	1.04
17	97	94	95	7	9	255	295	33	4576	66.8	0.92
18	97	95	94	7	22	249	288	36	3810	57.6	1.09
19	98	94	96	7	19	220	255	40	4182	58.7	1.04
20	95	96	97	7	7	131	151	46	1973	38.7	1.02
21	97	94	96	8	11	126	146	62.5	2202	41.5	1.01

SO_2 = oxygen saturation; interval= days between constant load tests; interval tt = days between both test types; IAT= indivdual anaerobic threshold; TTE= time to exhaustion; VO_2 top= highest test specific oxygen consumption; RER= respiratory exchange ratio

constant load test (placebo)

n	HR rest [bpm]	HR 5 min [bpm]	HR 10 min [bpm]	HR 15 min [bpm]	HR 20 min [bpm]	HR 25 min [bpm]	HR 30 min [bpm]	HR 35 min [bpm]	HF 40 min [bpm] n=15	HR 45 min [bpm] n=11	HR 50 min [bpm] n=7	HR top [bpm]
1	74	159	164	168	169	173	174	184				184
2	72	166	169	169	174	177	183	192	196	198		198
3	67	140	147	149	151	153	157	164	166			166
4	76	152	158	159	160	160	165	170	172	174	175	175
5	52	151	159	159	160	166	170	180	178	181	182	182
6	65	151	158	161	164	168	171	173	178	179	180	180
7	76	162	162	163	162	159	166	177	185	189		189
8	70	155	157	160	163	167	169	184	190	192	200	200
9	73	172	177	181	181	185	182	191	192	196		196
10	58	161	163	167	166	164	170	177	180			183
11	70	137	147	148	152	154	158	166				166
12	69	157	163	162	166	166	168	178	179	181	182	187
13	61	125	131	132	133	136	140	149	153	154	155	156
14	59	152	161	161	161	163	162	171				173
15	75	173	180	181	184	183	185	192				192
16	68	157	164	164	165	169	169	182				182
17	61	158	168	173	177	181	185	191				191
18	95	162	172	175	178	180	186	193	196			196
19	73	151	159	164	165	164	168	184	191			191
20	49	139	144	147	151	151	158	164	168	170		170
21	69	147	158	160	160	165	168	176	178	182	187	190

HR rest= heart rate at rest; HR top= highest test specific heart rate

constant load test (placebo)

n	Lac rest [mmol·l^{-1}]	Lac 5 min [mmol·l^{-1}]	Lac 10 min [mmol·l^{-1}]	Lac 15 min [mmol·l^{-1}]	Lac 20 min [mmol·l^{-1}]	Lac 25 min [mmol·l^{-1}]	Lac 30 min [mmol·l^{-1}]	Lac 35 min [mmol·l^{-1}]	Lac 40 min [mmol·l^{-1}] n=15	Lac 45 min [mmol·l^{-1}] n=11	Lac 50 min [mmol·l^{-1}] n=7	Lac top [mmol·l^{-1}]
1	0.7	3.6	3.9	4.3	4.8	5.0	5.0	8.8				8.8
2	0.5	3.6	2.8	2.9	2.7	3.4	3.3	6.9	9.0	10.5		10.5
3	0.5	2.9	3.2	3.8	3.9	3.6	3.9	5.7	6.5			6.5
4	0.9	3.0	2.6	2.5	2.4	2.5	2.4	4.4	4.8	5.7	6.1	6.1
5	0.5	4.2	4.4	4.5	4.7	4.7	4.9	7.6	9.8	10.7	10.8	10.6
6	1.1	3.4	3.2	3.4	3.4	4.0	4.5	6.1	8.0	9.8	11.2	11.2
7	0.7	5.9	5.7	5.6	5.2	4.7	4.5	7.2	9.5	11.7		11.7
8	0.7	1.6	1.4	1.4	1.9	1.8	1.7	3.5	4.4	4.8	6.5	6.5
9	0.5	3.3	3.8	4.0	3.6	3.4	3.2	6.0	6.9	8.2		8.2
10	1.0	3.5	2.9	2.3	2.0	2.0	2.1	3.6	4.6			5.7
11	0.7	3.9	3.2	3.0	3.1	3.2	2.9	5.2				5.2
12	1.2	3.9	3.1	3.1	2.7	3.2	2.4	6.0	6.1	6.6	7.1	8.8
13	0.6	3.8	3.4	2.6	2.6	2.5	2.6	4.3	5.1	5.1	5.6	6.1
14	0.6	4.6	4.8	4.4	8.5	4.5	6.6	8.6				8.5
15	0.5	2.9	2.6	2.4	2.7	2.5	2.6	3.9				3.9
16	0.5	4.2	3.4	3.4	3.1	2.8	2.5	4.6				4.6
17	0.9	4.1	3.7	4.1	5.9	5.9	5.8	8.1				8.1
18	0.5	6.1	6.0	5.2	5.5	6.0	6.4	10.8	12.9			12.9
19	0.5	4.8	4.2	4.0	3.5	2.7	2.6	5.9	9.5			9.5
20	0.5	4.5	3.9	2.9	2.6	2.5	2.6	4.4	5.0	5.4		5.4
21	0.7	2.9	2.0	1.5	1.4	1.4	1.3	2.1	2.3	2.5	2.3	2.3

Lac rest= lactate concentration at rest,
Lac top= highest test specific lactate concentration

constant load test (BICA)

n	ph pre	ph post	ph 5 min	ph 10 min	ph 15 min	ph 20 min	ph 25 min	ph 30 min	ph 35 min	ph 40 min	ph 45 min	ph 50 min	pH 2 min p
										n=15	n=11	n=7	
1	7.45	7.50	7.50	7.47	7.47	7.47	7.46	7.46	7.39				7.38
2	7.44	7.50	7.43	7.44	7.45	7.45	7.45	7.45	7.40	7.39	7.38		7.39
3	7.40	7.48	7.44	7.44	7.44	7.44	7.43	7.45	7.42	7.41			7.40
4	7.43	7.51	7.49	7.51	7.50	7.52	7.51	7.51	7.52	7.50	7.49	7.48	7.47
5	7.41	7.46	7.39	7.40	7.42	7.43	7.43	7.44	7.41	7.39	7.37	7.36	7.33
6	7.44	7.49	7.42	7.42	7.43	7.44	7.43	7.40	7.38	7.37	7.36	7.35	7.33
7	7.41	7.50	7.42	7.42	7.43	7.45	7.44	7.44	7.40	7.33	7.36		7.33
8	7.43	7.50	7.43	7.43	7.43	7.43	7.42	7.44	7.41	7.43	7.42	7.43	7.37
9	7.41	7.47	7.42	7.43	7.44	7.44	7.43	7.42	7.41	7.39	7.4		7.45
10	7.41	7.48	7.43	7.44	7.44	7.46	7.44	7.45	7.42	7.37			7.39
11	7.41	7.50	7.47	7.48	7.49	7.48	7.46	7.47	7.41				7.39
12	7.42	7.46	7.41	7.42	7.43	7.42	7.44	7.43	7.41	7.40	7.39	7.38	7.37
13	7.43	7.51	7.47	7.49	7.50	7.50	7.51	7.51	7.50	7.49	7.47	7.48	7.47
14	7.40	7.45	7.42	7.42	7.44	7.44	7.45	7.46	7.41				7.39
15	7.46	7.48	7.44	7.43	7.46	7.46	7.46	7.44	7.42				7.41
16	7.46	7.48	7.42	7.44	7.45	7.45	7.47	7.45	7.43				7.42
17	7.40	7.48	7.42	7.44	7.44	7.43	7.43	7.44	7.37				7.33
18	7.44	7.45	7.40	7.42	7.44	7.45	7.45	7.45	7.40	7.35			7.33
19	7.44	7.47	7.39	7.41	7.42	7.43	7.45	7.45	7.41	7.39			7.36
20	7.46	7.47	7.46	7.46	7.49	7.48	7.48	7.49	7.49	7.49	7.48		7.49
21	7.45	7.49	7.42	7.41	7.43	7.41	7.42	7.43	7.44	7.44	7.45	7.46	7.45

pre = pre drink; post = post drink; 2 min p = 2 min post exercise

constant load test (BICA)

n	BIC post [mmol·l^{-1}]	BIC 30 min [mmol·l^{-1}]	BIC 2 min p [mmol·l^{-1}]	BE post [mmol·l^{-1}]	BE 30 min [mmol·l^{-1}]	BE 2 min p [mmol·l^{-1}]	pCO_2 post [mmol·l^{-1}]	pCO_2 30 min [mmol·l^{-1}]	pCO_2 2 min p [mmol·l^{-1}]	pO_2 post [mmol·l^{-1}]	$pO_2$30 min [mmol·l^{-1}]	pO_2 2 min p [mmol·l^{-1}]
1	32	28	19	9	1	-1	42	33	34	82	68	92
2	32	29	20	8	5	-4	41	42	34	86	64	84
3	32	29	25	8	4	0	43	41	40	87	72	71
4	29	26	23	5	3	2	36	32	33	81	91	80
5	33	28	16	8	3	-7	46	41	32	60	71	74
6	32	25	15	7	-1	-8	42	39	30	78	69	75
7	32	28	18	8	3	-5	41	39	34	83	69	84
8	33	29	20	9	5	-4	42	44	37	86	68	91
9	31	27	23	7	3	0	44	41	34	88	67	88
10	33	29	11	8	3	-10	45	40	19	67	68	87
11	33	31	22	9	6	-2	45	40	37	82	66	83
12	30	27	20	6	3	-2	42	42	35	70	66	77
13	31	26	21	8	5	-1	40	35	29	76	68	75
14	32	27	22	7	3	-3	45	38	38	88	80	90
15	31	27	24	7	2	1	42	36	39	93	78	84
16	33	28	24	6	4	0	42	40	37	86	75	89
17	30	25	16	7	1	-9	42	35	31	84	75	87
18	30	27	16	7	2	-10	45	40	32	85	70	85
19	29	27	18	6	3	-4	40	39	32	93	69	81
20	30	27	23	6	4	0	41	35	30	84	71	95
21	30	29	28	6	4	0	40	43	42	79	71	81

post = post drink; 2 min p= 2 min post exercise; BIC = bicarbonate concentration; BE = base excess;
pCO_2 = partial pressure of carbon dioxide; pO_2 = partial pressure of oxygen

constant load test (BICA)

n	SO_2 post [mmol·l^{-1}]	SO_2 30 min [mmol·l^{-1}]	SO_2 2 min [mmol·l^{-1}]	TTE [min]	VO_2 top [ml·min^{-1}]	VO_2 top [ml·min^{-1}·kg^{-1}]	RER
1	97	94	98	32.5	5018	64.3	1.10
2	97	93	96	61	5062	73.9	0.98
3	97	95	94	56	3722	53.9	1.03
4	97	98	96	60	2778	55.6	0.93
5	92	94	94	67	3817	60.6	1.13
6	96	94	94	64	3368	57.1	0.99
7	97	94	96	44	4973	64.6	1.03
8	97	94	97	63	4426	57.5	1.10
9	97	93	97	61.5	5379	74.7	0.97
10	94	94	99	38	4572	63.5	1.07
11	97	94	96	44	3982	56.1	1.03
12	95	94	95	50	4301	60.6	1.00
13	96	94	96	57.5	3124	42.2	1.03
14	97	96	97	36.5	3765	57.9	1.03
15	97	96	94	32.5	4185	50.7	0.96
16	97	95	97	36	4040	58.6	1.06
17	97	95	96	40	4477	64.9	1.06
18	97	94	96	40	4015	62.2	1.08
19	97	94	95	45	4278	59.4	1.02
20	97	95	98	61.3	2127	42.1	1.06
21	96	94	96	50	2524	48.5	1.01

SO_2 = oxygen saturation; IAT= indivdual anaerobic threshold; TTE= time to exhaustion;
VO_2 top= highest test specific oxygen consumption; RER= respiratory exchange ratio

constant load test (BICA)

n	HR rest [bpm]	HR 5 min [bpm]	HR 10 min [bpm]	HR 15 min [bpm]	HR 20 min [bpm]	HR 25 min [bpm]	HR 30 min [bpm]	HR 35 min [bpm]	HF 40 min [bpm] n=15	HR 45 min [bpm] n=11	HR 50 min [bpm] n=7	HR top [bpm]
1	75	161	168	169	173	174	180	186				186
2	76	163	168	170	171	175	183	186	191	192		195
3	71	141	148	152	152	151	155	163	168			173
4	65	150	154	156	155	156	158	164	165	166	167	167
5	61	148	155	156	158	160	161	172	175	177	176	181
6	75	140	151	157	160	165	167	172	173	174	177	178
7	71	158	161	164	160	168	171	175	182	184		184
8	70	163	166	169	175	172	172	182	185	189	189	200
9	83	173	177	183	183	179	182	191	190	189		195
10	51	164	168	170	171	174	177	188	189			189
11	70	145	155	156	159	161	164	170				174
12	64	152	153	154	155	162	164	170	175	177	179	179
13	60	133	135	138	139	139	143	149	151	152	153	156
14	67	157	162	164	163	163	163	172				172
15	70	178	187	190	191	193	194	200				200
16	83	162	171	173	169	175	181	187				187
17	71	157	171	171	175	177	181	187				192
18	76	160	168	172	174	178	180	189	192			192
19	75	151	157	160	160	162	167	179	185			189
20	54	138	138	142	145	148	150	158	159	162		164
21	65	155	166	166	169	175	182	189	188	190	190	190

HR rest= heart rate at rest; HR top= highest test specific heart rate

constant load test (BICA)

n	Lac rest [mmol·l^{-1}]	Lac 5 min [mmol·l^{-1}]	Lac 10 min [mmol·l^{-1}]	Lac 15 min [mmol·l^{-1}]	Lac 20 min [mmol·l^{-1}]	Lac 25 min [mmol·l^{-1}]	Lac 30 min [mmol·l^{-1}]	Lac 35 min [mmol·l^{-1}]	Lac 40 min [mmol·l^{-1}] n=15	Lac 45 min [mmol·l^{-1}] n=11	Lac 50 min [mmol·l^{-1}] n=7	Lac top [mmol·l^{-1}]
1	1.6	4.7	7.2	7.6	7.9	7.8	8.0	10.7				10.7
2	0.7	3.9	3.7	3.4	3.1	2.8	3.0	5.7	7.5	8.7		10.5
3	0.6	3.4	3.9	3.9	3.9	4.1	3.9	6.1	7.5			8.0
4	1.8	3.0	2.8	2.8	2.7	2.6	2.3	3.4	4.1	4.6	4.8	4.9
5	0.8	5.6	5.9	5.6	5.4	5.0	4.7	7.2	8.9	10.8	11.4	13.2
6	0.9	4.5	3.8	3.7	4.1	4.3	4.7	6.4	8.0	9.0	10.0	13.5
7	1.3	5.3	5.8	5.9	6.0	6.0	6.0	8.6	11.3	13.7		13.7
8	1.1	3.3	2.9	3.2	3.4	3.5	3.2	4.3	5.2	5.6	5.8	8.6
9	0.9	3.9	4.3	4.2	4.1	3.9	3.5	6.3	7.9	7.7		7.2
10	1.0	4.0	3.8	3.7	3.8	4.0	4.0	7.7	9.6			9.6
11	1.0	4.3	3.5	3.3	5.0	4.2	4.7	7.9				10.6
12	0.6	3.3	3.0	2.4	2.5	2.6	3.1	5.1	6.4	7.2	8.4	8.4
13	0.9	4.2	4.0	3.6	3.6	3.3	3.3	5.0	6.0	5.6	6.6	7.8
14	0.5	4.4	4.7	4.0	4.0	4.0	4.1	6.4				6.4
15	0.5	3.9	4.0	3.9	3.8	4.2	3.3	4.9				4.9
16	0.8	3.8	3.4	3.3	3.1	3.2	3.1	5.7				5.7
17	0.9	6.2	6.4	7.0	7.6	7.8	7.5	11.7				14.0
18	0.6	6.3	6.1	5.9	5.4	5.4	5.6	9.3	12.3			12.3
19	0.9	5.9	5.7	5.1	4.5	4.2	4.0	7.8	9.8			11.6
20	0.6	4.3	3.9	3.2	3.4	3.4	3.1	4.1	4.6	5.1		5.7
21	0.8	2.7	2.4	2.0	1.8	1.9	1.8	3.4	3.8	4.1	3.8	3.8

Lac rest= lactate concentration at rest,
Lac top= highest test specific lactate concentration
