# Supplementary material for: Effects of Sodium Bicarbonate on High-Intensity Endurance Performance in Cyclists: A Double-Blind, Randomized Cross-Over Trial
Source: PLoS One. 2014 Dec 10;9(12):e114729. doi: 10.1371/journal.pone.0114729 (PMC4262454; doi:10.1371/journal.pone.0114729)
Supplement: S1 Protocol — Study protocol. The original study protocol has been carefully translated into English language. (PDF) [file pone.0114729.s003.pdf]

Trial study protocol approved by the local ethics committee

(Aerztekammer des Saarlandes, Saarbruecken, Germany)

Effects of sodium bicarbonate on performance in endurance athletes (cyclists)

Purpose:

Does buffering of metabolic acids by sodium bicarbonate lead to performance improvements in an incremental cycle test and or open-end-test?

Subjects:

well-trained triathletes or cyclists; age > 18 years

General design:

Initially, subjects discuss their medical history and receive a physical examination, an anthropometric measurement, an ECG, a pulmonary function test with measurement of inspiratory pressures ($PI_{max}/P_{0.1}$) and a venous blood sample (to rule out kidney diseases and mineral imbalances).
With an interval of one week two exhaustive cycle ergometries (incremental cycle test) with spriroergometric measurement and determination of individual anaerobic threshhold (IAT) will be conducted. In a double-blind, randomized manner subjects ingest two hours prior to each test a sodium bicarbonate solution (0.3g/kg solved in 0.7 litre water, ingested within 60 min) or an equal volume of a placebo solution.
Another one or two weeks later subjects will perform two cycle ergometries (open-end-test), in which they exercise for 30 min at a workload equal to 95% of IAT followed by 110% IAT until exhaustion. For this, IAT from the incremental cycle test with placebo is used. Again, subjects ingested a solution of sodium bicarbonate or placebo prior to testing. Blood gas analyses from the hyperemized earlobe will happen prior to ingestion of the test substances, before testing, every 5 min during exercise and post exercise. Additionally, blood lactate is analyzed every 5 min during exercise, at exhaustion and 1, 3, 5 min post exercise.
During the test period subjects are not allowed to take part in a competition or other season highlights. On the day before testing subjects are instructed to exercise at low intensity, if necessary, and to eat sufficiently. On the examination day, a sufficient dietary intake should be followed up to three hours before testing.

Exclusion criteria:

Acid-base or electrolyte imbalances; cardiovascular diseases or doubts about full physical fitness; maximal performance < 5.0 W/kg.

Hypotheses:

- During the incremental cycle test sodium bicarbonate leads to
  - no alteration of the individual anaerobic threshold (IAT)
  - increase of maximal performance
- During the open-end-test sodium bicarbonate leads to:
  - prolonged cycling time and accordingly later onset of fatigue

Sample size:
Sample size was estimated with the program G*Power (version 3.1.5) using the following parameters: effect size to be detected 0.6, alpha error probability 0.05, 1- β error probability 0.8; 1-tailed testing. The calculated sample size of 19 was rounded up to 20.

Purposes:

Effects of sodium bicarbonate on maximal performance, IAT, exercise lactate curves and peak oxygen consumption in the incremental cycle test, performance in the open-end-test.

1. Formalities:

1.1 **Designation of operation:**
Effects of sodium bicarbonate on performance in endurance athletes

1.2 **Name of the responsible project leader and the involved physicians:**
Prof. Dr. med. Tim Meyer, Dr. med. Ulf Such

1.3 **Type and number of inspection authorities and names of physicians in the case of multi-center-studies**
Reserved.

1.4 **Name and address of the sponsor**
Reserved.

1.5 **Has there ever been such a request to another ethics committee ?**
No.

1.5.1 **To which ?**
Reserved.

1.5.2 **Submission of the vote including the restrictions of this ethics committee including the potentially exchanged correspondence.**
Reserved.

2. Description and scientific justification of the project

2.1 Explanation of the objective

It is well documented that the intake of sodium bicarbonate may improve performance mainly in anaerobic exercise lasting 1 to 7 minutes [1,2]. Though considerably less research has been done, endurance performance seems not to be influenced [3].

The proposed study aims to examine whether anaerobic endurance performance (cycle ergometer), after exercising at intensity just below the individual anaerobic threshold, may be improved through prior ingestion of sodium bicarbonate. It's conceivable that the buffering capacity will already be exhausted, when anaerobic exercise begins. By analyses of acid-base balance we will be able to document accurate courses of pH and buffering capacity

2.2 Description of the current state of knowledge [2], [4-6]

There is consensus that muscular fatigue at high-intensity exercise is at least in part due to the decrease of intramuscular pH. Anaerobic energy supply leads to accumulation of acids (especially lactic acid), which dissociates into lactate ions and hydrogen ions, thereby decreasing the pH of muscle and blood. The activity of intracellular enzymes (e.g. the key enzyme of glycolysis: phosphofructokinase) is pH-dependent and decreases with increasing acidosis. Hydrogencarbonate is an important extracellular buffer. Increased extracellular buffering capacity will lead to accelerated efflux of intracellular accumulating hydrogen ions, so that pH could be kept constant at similar accumulation of acids and accordingly similar metabolic activity.

2.3 Results of the pharmacological-toxicological pre-examination (laboratory tests and animal experiments)

Reserved.

2.4 Submission of the entire test plan

Subjects:
well-trained triathletes or cyclists; age > 18 years

General design:
Initially subjects discuss their medical history and receive a physical examination, an anthropometric measurement, an ECG, a pulmonary function test with measurement of inspiratory pressures (PI_{max}/$P_{0.1}$) and a venous blood sample (to rule out kidney diseases and mineral imbalances).

In an interval of one week two exhaustive cycle ergometries (incremental cycle test) with spriroergometric measurement and determination of individual anaerobic threshhold (IAT) will be conducted. In a double-blind, randomized manner subjects ingested two hours prior to each testing a sodium bicarbonate solution (0.3g/kg solved in 0.75 litre water, ingested within 60 min) or an equal volume of a placebo solution.

Another one or two weeks later subjects will perform two cycle ergometries (open-end-test), in which they exercise for 30 min at an workload equal to 95% of IAT followed by 110% IAT until exhaustion. For this, IAT from the incremental cycle test with placebo is used. Again, subjects ingested a solution of sodium bicarbonate or placebo prior to testing. Blood gas analyses

from the hyperemized earlobe follow prior to ingestion of test substances, before testing and post exercise. Additionally, blood lactate was analyzed post exercise.

2.5 **Envisaged overall duration of examination**
6 months.

2.6 **Justification for the need of studies on humans**
An animal model, which is predictive to human outcomes, is not available for this scientific question.

2.6.1 **Study on healthy subjects ?**
Yes.

2.6.2 **Study on patients ?**
No.

2.6.3. **Inclusion criteria**
1.) Age of majority
2.) Good performance capacity with maximal performance of at least 5 Watt/kg in cyclists in the incremental cycle test.

2.6.4 **Exclusion criteria**
1.) Acid-base or electrolyte imbalances
2.) Cardiovascular diseases or doubts about full physical fitness
3.) Current intake of medication

2.6.5 **Intermediate exclusion criteria**
Acute injuries or diseases.

2.6.6 **Concurrent medication**
Reserved.

2.6.7 **Indication of side effects**
Sodium bicarbonate may decrease the blood potassium level. By means of blood samples a preexisting hypokalaemia can be ruled out. Sodium bicarbonate is freely available, non-pharmacy-restricted and completely unproblematic concerning doping guidelines.

2.6.8 **Statement to possible risks and side effects (including the not yet described)**
Though no complications are expected, complete emergency equipment is available.

2.6.9 **Discontinuation criteria**
1.) The subject's decision to withdraw from the study
2.) (gastrointestinal) intolerance of sodium bicarbonate
3.) Health problems, which manifest in the course of the study

2.7.0 **Designation of advisory and control commission**
Reserved.

3 References:

1. Lindermann JK GK (1994) The effects of sodium bicarbonate ingestion on exercise performance. Sports Med 18: 75-80.
2. McNaughton LR, Siegler J, Midgley A (2008) Ergogenic effects of sodium bicarbonate. Curr Sports Med Rep 7: 230-236.
3. George KP, MacLaren DP (1988) The effect of induced alkalosis and acidosis on endurance running at an intensity corresponding to 4 mM blood lactate. Ergonomics 31: 1639-1645.
4. Lindermann JK FT (1991) Sodium bicarbonate ingestion and exercise performance. An update. Sports Med 11: 71–77.
5. Kemp G, Boning D, Beneke R, Maassen N (2006) Explaining pH change in exercising muscle: lactic acid, proton consumption, and buffering vs. strong ion difference. Am J Physiol Regul Integr Comp Physiol 291: R235-237; author reply R238-239.
6. Zinner C, Wahl P, Achtzehn S, Sperlich B, Mester J (2011) Effects of bicarbonate ingestion and high intensity exercise on lactate and H(+)-ion distribution in different blood compartments. Eur J Appl Physiol 111: 1641-1648.
