# Supplementary material for: Effects of Sodium Bicarbonate on High-Intensity Endurance Performance in Cyclists: A Double-Blind, Randomized Cross-Over Trial
Source: PLoS One. 2014 Dec 10;9(12):e114729. doi: 10.1371/journal.pone.0114729 (PMC4262454; doi:10.1371/journal.pone.0114729)
Supplement: S2 Protocol — Study protocol in original language (German). (PDF) [file pone.0114729.s004.pdf]

Studienprotokoll anerkannt durch die lokale Ethikkommission

(Aerztekammer des Saarlandes, Saarbruecken, Deutschland)

Effekte einer Einnahme von Natriumhydrogenkarbonat auf die Leistungsfähigkeit von Ausdauersportlern (Radfahrer)

Untersuchungsziel:

Führt eine Pufferung von metabolisch anfallenden Säuren durch Natriumbikarbonat zu einem Anstieg der Leistungsfähigkeit im Stufen- und/oder im Open-End-Test

Probanden:

Gut trainierte Triathleten oder Radfahrer; Alter > 18 Jahre

Allgemeiner Untersuchungsablauf:

Die Probanden erhalten zu Beginn eine Anamnese und eine körperliche Untersuchung, eine Messung anthropometrischer Größen, ein EKG, eine Lungenfunktionsprüfung mit Messung der Inspirationsdrücke (PI_{max}/$P_{0.1}$) und eine venöse Blutentnahme (zum Ausschluss von Nierenerkrankungen oder Störungen des Mineralhaushalts).
In einem Abstand von einer Woche erfolgen zwei ausbelastende Fahrradergometrien (Stufenprotokoll) mit Spiroergometrie und Bestimmung der individuellen anaeroben Schwelle (IAS). Doppelblind und randomisiert werden die Probanden jeweils einmal zwei Stunden vor Testbeginn eine Lösung mit Natriumhydrogenkarbonat (0,3g/kg gelöst in 0,7 Liter Wasser, getrunken innerhalb 60 min) oder eine Placebolösung der gleichen Menge trinken.
Weitere ein bzw. zwei Wochen später werden die Probanden zwei Fahrradergometrien (Open-End-Test) durchführen, bei denen sie 30 min mit der Leistung von 95% der IAS fahren und anschließend mit 110% der IAS bis zur Erschöpfung. Hierzu wurde die IAS verwendet, die unter der Placebolösung im vorangegangenen Stufentest bestimmt wurde. Auch vor diesen Tests trinken die Probanden erneut wieder die Natriumhydrogenkarbonat- oder eine Placebolösung. Blutgasanalysen aus dem hyperämiserten Ohrläppchen erfolgen vor dem Trinken der Testlösung, vor Testbeginn, alle 5 Minuten während der Belastung und in der Nachbelastungsphase. Zusätzlich werden Laktatwerte alle 5 Minuten während der Belastung und direkt bei Belastungsabbruch sowie in den Minuten 1, 3, 5 in der Nachbelastungsphase gemessen.
In den Wochen der Untersuchung dürfen die Probanden keine Wettkämpfe oder Saisonhöhepunkte haben. Am Vortag einer Untersuchung sollen sie Sport höchstens mit leichter Intensität ausüben und ausreichend gegessen haben. Am Untersuchungstag selbst sollte ebenfalls ausreichend Nahrung aufgenommen werden, allerdings nicht mehr in den letzten drei Stunden vor Beginn einer Fahrradergometrie.

Ausschlusskriterien:

Störungen des Elektrolyt- oder Säure-Basen-Status; Herzkreislauferkrankungen oder Zweifel an der Sporttauglichkeit; Maximalleistung < 5.0 W/kg.

Hypothesen:

Natriumhydrogenkarbonat führt zu
- im Stufentest:
    - zu keiner Änderung der individuellen anaeroben Schwelle (IAS)
    - zu einer Zunahme der maximalen Leistung
- im Open-End-Test:
    - zu einer längeren Fahrleistung bzw. einem späteren Belastungsabbruch

Stichprobengröße:
Die vorraussichtliche Größe der Stichprobe wurde mit dem Programm G*Power (Version 1.3.5) bestimmt. Zur Berechnung wurden folgende Parameter verwendet: Effektgröße 0,6; alpha-Fehler Wahrscheinlichkeit 0,05; 1- β-Fehler Wahrscheinlichkeit 0,8; einseitiger Test. Die kalkulierte Stichprobe von 19 wurde auf 20 aufgerundet.

Untersuchungsziele:

Einfluss der Natriumhydrogenkarbonat-Einnahme auf maximale Leistung, IAS, Laktat-Leistungs-Kurve, und maximale Sauerstoffaufnahme im Stufentest sowie die Leistung im Open-End-Test.

1. Formales

1.1 Bezeichnung des Vorhabens
Effekte einer Einnahme von Natriumhydrogenkarbonat auf die Leistungsfähigkeit von Ausdauersportlern

1.2 Name des verantwortlichen Leiters und seiner mitbetreuenden Ärzte
Prof. Dr. med. Tim Meyer, Dr. med. Ulf Such

1.3 Art und Zahl der Prüfstellen und Namen der beteiligten Ärzte bei Multicenterstudien
Entfällt.

1.4 Name und Adresse des Sponsors
Entfällt.

1.5 Wurde schon bei einer anderen Ethikkommission ein diesbezüglicher Antrag gestellt ?
Nein.

1.5.1 Bei welcher ?
Entfällt.

1.5.2 Vorlage des Votums einschl. der von dieser Ethikkommission gemachten Auflagen einschl. des evtl. geführten Schriftwechsels

Entfällt.

2. Beschreibung und wissenschaftliche Begründung des Projekts

2.1 Erläuterung des Studienziels

Es ist durch zahlreiche Studien abgesichert, dass die Einnahme von Natriumhydrogenkarbonat bei überwiegend anaeroben Belastungen, die zwischen ein und sieben Minuten dauern, leistungssteigernd wirken kann [1,2]. Obgleich sehr viel weniger untersucht, scheinen reine Ausdauerleistungen nicht beeinflussbar zu sein [3].

Mit der beantragten Studie wollen wir herausfinden, ob im Anschluss an eine Ausdauerleistung (Fahrradergometrie) knapp unterhalb der individuellen anaeroben Schwelle, eine Leistungssteigerung durch vorherige Natriumhydrogenkarbonat-Einnahme im anaeroben Bereich möglich ist. Es ist denkbar, dass die Pufferkapazität durch die vorangegangene Belastung bereits erschöpft ist, wenn die anaerobe Belastung einsetzt. Durch Analysen des Säure-Basen-Haushalts während der Belastung werden wir einen genauen Verlauf von pH-Wert und Pufferkapazität dokumentieren können.

2.2 Darstellung des bisherigen Wissensstandes [2], [4-6]

Es herrscht Konsens, dass die Erschöpfung der Arbeitmuskulaturbei hohen Belastungen zumindest teilweise durch einen Abfall des intramuskulären pH-Werts verursacht ist. Durch die anaerobe Energiebereitstellung fallen Säuren an (insbesondere Milchsäure), die zu Laktat und Wasserstoff dissoziieren und somit den pH im Muskel und Blut senken. Die Aktivität intrazellulärer Enzyme (z.B. des „Schrittmacherenzyms“ der Glykolyse: Phosphofruktokinase) ist pH-abhängig und sinkt mit zunehmender Azidose. Hydrogenkarbonat ist ein wichtiger extrazellulärer Puffer. Bei großer extrazellulärer Pufferkapazität wird der Ausstrom der intrtazellulär anfallenden Wasserstoffionen beschleunigt, sodass theoretisch bei gleicher Stoffwechselaktivität bzw. Säureanfall der pH länger konstant gehalten werden kann.

2.3 Ergebnisse der pharmakologisch-toxikologischen Vorprüfungen (Labor- und Tierversuche)

Entfällt.

2.4 Vorlage des gesamten Prüfplans in deutscher Sprache

<u>Probanden:</u>
Gut trainierte Triathleten oder Radfahrer; Alter > 18 Jahre

<u>General design:</u>
Die Probanden erhalten zu Beginn eine Anamnese und eine körperliche Untersuchung, eine Messung anthropometrischer Größen, ein EKG, eine Lungenfunktionsprüfung mit Messung der Inspirationsdrücke ($PI_{max}/P_{0.1}$) und eine venöse Blutentnahme (zum Ausschluss von Nierenerkrankungen oder Störungen des Mineralhaushalts).

In einem Abstand von einer Woche erfolgen zwei ausbelastende Fahrradergometrien (Stufenprotokoll) mit Spiroergometrie und Bestimmung der individuellen anaeroben Schwelle (IAS). Doppelblind und randomisiert werden die Probanden jeweils einmal zwei Stunden vor Testbeginn eine Lösung mit Natriumhydrogenkarbonat (0,3g/kg gelöst in 0,7 Liter Wasser, getrunken innerhalb 60 min) oder eine Placebolösung der gleichen Menge trinken.
Weitere ein bzw. zwei Wochen später werden die Probanden zwei Fahrradergometrien (Open-End-Test) durchführen, bei denen sie 30 min mit der Leistung von 95% der IAS fahren und anschließend mit 110% der IAS bis zur Erschöpfung. Hierzu wurde die IAS verwendet, die unter der Placebolösung im vorangegangenen Stufentest bestimmt wurde. Auch vor diesen Tests trinken die Probanden erneut wieder die Natriumhydrogenkarbonat- oder eine Placebolösung. Blutgasanalysen aus dem hyperämiserten Ohrläppchen erfolgen vor dem Trinken der Testlösung, vor Testbeginn und in der Nachtestphase. Zusätzlich werden Laktatwerte in der Erholungsphase gemessen.

2.5 **Vorgesehene Gesamtdauer der Untersuchungen**
6 Monate

2.6 **Begründung der Notwendigkeit von Studien an Menschen**
Ein Tiermodell, das eine Übertragbarkeit auf den Menschen zulassen würde, steht für diese Fragestellung nicht zur Verfügung.

2.6.1 **Studie an gesunden Probanden ?**
Ja.

2.6.2 **Studie an Patienten ?**
Nein.

2.6.3. **Einschlusskriterien**
1.) Volljährigkeit
2.) Gutes Leistungsvermögen mit einer Maximalleistung von mindestens 5 Watt/kg bei Radfahrern im Stufentest

2.6.4 **Ausschlusskriterien**
1.) Störungen des Elektrolyt- oder Säure-Basen-Status
2.) Herzkreislauferkrankungen oder Zweifel an der Sporttauglichkeit
3.) Aktuelle Einnahme von Medikamenten

2.6.5 **Zwischenausschlusskriterien**
Akute Verletzungen oder Erkrankungen

2.6.6 **Begleitmedikation**
Entfällt.

2.6.7 **Eingehen auf etwaige Kontraindikationen**
Natriumhydrogenkarbonat kann den Blutkaliumspiegel senken. Mittels Blutentnahme werden vorbestehende Hypokaliämien ausgeschlossen.

Natriumhydrogenkarbonat ist frei verkäuflich, nicht apothekenpflichtig und hinsichtlich der Dopingrichtlinien völlig unproblematisch.

2.6.8 **Stellungnahme zu möglichen, auch bisher noch nicht beschriebenen Risiken und Nebenwirkungen**
Obwohl kaum Komplikationen zu erwarten sind, steht eine komplette Notfallausrüstung bei den Belastungsuntersuchungen bereit.

2.6.9 **Abbruchkriterien**
1.) Rücktrittswunsch des Probanden
2.) (gastrointestinal) Unverträglichkeit des Natriumhydrogenkarbonats
3.) Gesundheitliche Probleme, die sich im Laufe der Studie manifestieren

2.7.0 **Nennung der Begleit- und Kontrollkommission.**
Reserved.

3 References:

1. Lindermann JK GK (1994) The effects of sodium bicarbonate ingestion on exercise performance. Sports Med 18: 75-80.
2. McNaughton LR, Siegler J, Midgley A (2008) Ergogenic effects of sodium bicarbonate. Curr Sports Med Rep 7: 230-236.
3. George KP, MacLaren DP (1988) The effect of induced alkalosis and acidosis on endurance running at an intensity corresponding to 4 mM blood lactate. Ergonomics 31: 1639-1645.
4. Lindermann JK FT (1991) Sodium bicarbonate ingestion and exercise performance. An update. Sports Med 11: 71–77.
5. Kemp G, Boning D, Beneke R, Maassen N (2006) Explaining pH change in exercising muscle: lactic acid, proton consumption, and buffering vs. strong ion difference. Am J Physiol Regul Integr Comp Physiol 291: R235-237; author reply R238-239.
6. Zinner C, Wahl P, Achtzehn S, Sperlich B, Mester J (2011) Effects of bicarbonate ingestion and high intensity exercise on lactate and H(+)-ion distribution in different blood compartments. Eur J Appl Physiol 111: 1641-1648.
